# Induction of Expression of p75 Neurotrophin Receptor Intracellular Domain Does Not Induce Expression or Enhance Activity of Mitochondrial Complex II

**DOI:** 10.1155/2016/8752821

**Published:** 2015-11-10

**Authors:** Yaoli Pu Yang, Louis Lotta, Gisela Beutner, Xingguo Li, Nina F. Schor

**Affiliations:** Department of Pediatrics, University of Rochester School of Medicine and Dentistry, Rochester, NY 14642, USA

## Abstract

Fenretinide is a chemotherapeutic agent in clinical trials for the treatment of neuroblastoma, among the most common and most deadly cancers of childhood. Fenretinide induces apoptosis in neuroblastoma cells through accumulation of mitochondrial reactive oxygen species released from Complex II. The neurotrophin receptor, p75NTR, potentiates this effect. The signaling activity of p75NTR is dependent upon its cleavage to its intracellular domain, p75ICD, trafficking of p75ICD to the nucleus, and functioning of p75ICD as a transcription factor. Mitochondrial Complex II comprises 4 subunits, all of which are encoded by nuclear DNA. We therefore hypothesized that the fenretinide-potentiating effects of p75NTR are the result of transcriptional enrichment of Complex II by p75ICD. However, the present studies demonstrate that neither induced expression of p75ICD or its active fragments nor overexpression of p75NTR results in altered expression or activity of Complex II.

## 1. Introduction

Neuroblastoma is the most common extracranial solid tumor of childhood. It derives from the neural crest and commonly presents clinically in the adrenal gland or sympathetic chain. Fenretinide, a retinoic acid derivative, is under active clinical investigation for the treatment of neuroblastoma. Unlike all-trans retinoic acid (ATRA), which is used to induce cellular differentiation in the treatment of cancer, fenretinide is known to cause apoptosis through generation of mitochondrial reactive oxygen species thought to “leak” from Complex II [1] and differs structurally from ATRA by only a hydroxyphenyl group. A recent phase II trial of 65 patients did not meet criteria for clinical efficacy [2] due to low bioavailability of the drug. However, there are now ongoing phase I clinical trials for a new formulation [3] as well as other drug delivery systems developing in the pipeline [4].

Our previous studies [1, 5] examined the dependence of fenretinide efficacy on components of the p75NTR proapoptotic signaling pathways and demonstrated enhancement of these pathways and attenuation of antiapoptotic pathways in the presence of p75NTR expression. However, the mechanism by which p75NTR enhances the accumulation of reactive oxygen species from Complex II in the mitochondria is not known. The presently described studies test the hypothesis that the intracellular domain of p75NTR (p75ICD), a transcription factor, enhances the expression and/or enzymatic activity of Complex II.

## 2. Materials and Methods

### 2.1. Cell Lines

We tested our hypothesis that p75NTR expression and cleavage to p75ICD alter expression and activity of mitochondrial Complex II by inducing p75ICD expression in NIH 3T3 fibroblasts because these cells are p75NTR- (and, therefore, p75ICD-) negative in their native state. NIH 3T3 cells were used after transfection with either a V5 tag-containing plasmid (mock) or a p75ICD plasmid construct containing the V5 tag. In addition, induction of expression of p75ICD, p75ICD lacking the chopper domain, or p75NTR death domain in NIH 3T3 cells was performed by transfection as we have previously described [6]. Briefly, full-length p75ICD and Chopper- or death domain-deleted p75ICD constructs were cloned with a V5 epitope into the pBig2i-IRES-eGFP doxycycline- (Dox-; Sigma-Aldrich, St. Louis, MO) regulated plasmid (Invitrogen, Carlsbad, CA). Plasmids were transfected into NIH 3T3 cells using Lipofectamine 2000 (Invitrogen) and stable lines were selected for resistance to Hygromycin (400 mg/mL; Invitrogen). Construct inducibility was verified by fluorescence microscopy for GFP expression and Western blot analysis for p75ICD fragment expression after Dox exposure for 36–48 h. Individual GFP-positive and GFP-negative clones were isolated for study.

In addition, in follow-up of our studies of p75NTR and fenretinide in SH-EP1 human neuroblastoma cells [1, 5], we studied p75ICD nuclear translocation in SH-EP1 cells transfected with a p75NTR expression construct or the corresponding empty vector as we described in the prior publications. Briefly, we used the forward ATGTGGAACAGCTGCAAATAAACAAGG and reverse CACAGGGGACGTGGCAGTGGAC primers to amplify p75ICD. The p75ICD was amplified by RT-PCR and cloned in frame with V5 epitope into pcDNA3.1 TOPO V5 His plasmid (Invitrogen). The successful cloning of p75ICD plasmid was verified by sequencing. Cells were then plated at 70% confluency 24–48 h prior to transfection; 2 × 10^6^ cells were trypsinized and the supernatant was centrifuged and removed. The pellet was resuspended in a 100 *μ*L mixture of Nucleofector solution (82%) and supplement solution (18%) and added to the cuvette followed by nucleofection using the corresponding program in the Nucleofector II machine according to the manufacturer's instructions (Amaxa). The transfected cells were plated in 6 cm dishes and treated with the resistance antibiotic (G418, 500 *μ*g/mL) to create stably transfected cells.

### 2.2. Chemicals and Reagents

Fenretinide was obtained from Sigma Chemical Corp. (St. Louis, MO). Antibody to the succinate dehydrogenase (Complex II) A subunit (SDH A) was obtained from Abcam (Cambridge, MA). Antibodies for p75NTR intracellular epitopes were obtained from Promega (Madison, WI).

### 2.3. MTS Assay

After treatment of cells with H_2_O_2_ or fenretinide (72 h), 20 *μ*L of CellTiter 96 AQueous One Solution Reagent (Promega) was added to each well of the 96-well assay plate containing the samples in 100 *μ*L of culture medium. The plate was incubated at 37°C for 1 h in a humidified, 5% CO_2_ atmosphere before reading the optical density of each well at 490 nm (OD_490 nm_) in a plate reader. Results were expressed as percent cell survival relative control sister cultures treated with vehicle alone.

### 2.4. Determination of Complex II Enzymatic Activity

Enzymatic function studies were done on NIH 3T3 cells with stably induced p75ICD or p75ICD fragment expression and mock-transfected control cells. Briefly, the enzymatic activity of Complex II is measured as succinate ubiquinone oxidoreductase. In this test, succinate is oxidized to fumarate and ubiquinone_1_ × H_2_, and the latter is the substrate for the artificial electron acceptor dichlorophenolindophenol (DCPIP). The resulting change is measured at 600 nm and the extinction coefficient for DCPIP is 19.1 mM^−1^ cm^−1^ [7]. Protein concentrations of each of the Complex II subunits (succinate dehydrogenase (SDH) A, B, C, and D) were determined using Western blotting and compared between p75ICD-expressing and mock-transfected control cells. RNA levels of Complex II subunits were assessed and compared using qRT-PCR with primers obtained from GeneCopoeia (Rockville, MD).

## 3. Results

### 3.1. Demonstration of Function of p75ICD and p75NTR in Transfected NIH 3T3 and SH-EP1 Cells, Respectively

We have previously demonstrated that p75NTR has prooxidant activity and that this activity is fully retained in p75ICD in SH-EP1 and NIH 3T3 cells [1, 6]. In an effort to demonstrate production of functional protein in our transfected NIH 3T3 and SH-EP1 cells, we have therefore compared p75NTR- or p75ICD-transfected cells with their mock-transfected counterparts relative to resistance to induction of cell death by oxidant stress.  Table 1 shows that transfection with expression constructs for either p75NTR or p75ICD resulted in potentiation of the cytocidal effects of H_2_O_2_ or fenretinide in NIH 3T3 or SH-EP1 cells, respectively.

### 3.2. Nuclear Trafficking of p75ICD

**Figure 1:**
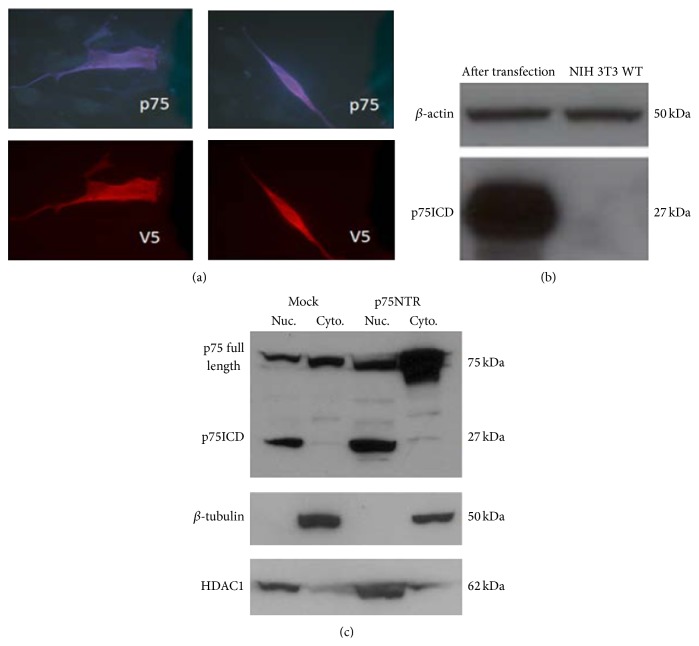
Subcellular localization of p75NTR and p75ICD in transfected NIH 3T3 and SH-EP1 cells. (a) Immunohistochemical staining with antibodies that stain p75ICD (blue) and its V5 tag (red) demonstrates that p75ICD is present in the nuclei of the cells. Fluorescence microscopy, 100x magnification. (b) Demonstration by Western blot of expression of p75ICD in transfected NIH 3T3 cells. (c) Demonstration by Western blot that transfection of SH-EP1 human neuroblastoma cells with an expression construct for p75NTR results in p75NTR in the nucleus and (predominantly) cytoplasm and p75ICD only in the nucleus.

Cells were fractionated by differential centrifugation prior to Western blotting to determine the compartments in which p75NTR and p75ICD were contained. Confirmation of the identities of the nuclear and cytoplasmic compartments was performed by blotting the membranes for histone deacetylase 1 and *β*-tubulin, respectively. Immunohistochemical staining and fluorescence microscopy were also performed with antibodies to p75ICD and V5. Figures 1(a) and 1(b) demonstrate in NIH 3T3 cells that p75ICD transfection and consequent cytoplasmic production of p75ICD results in cells that contain p75ICD in both the cytoplasm and the nucleus, implying that p75ICD traffics to the nucleus.  Figure 1(c) demonstrates in SH-EP1 human neuroblastoma cells that, after induced overexpression of p75NTR, p75ICD is found only in the nucleus. This is consistent with the role of p75ICD as a transcription factor.

### 3.3. Effect of p75ICD Expression on Expression of Complex II

**Figure 2:**
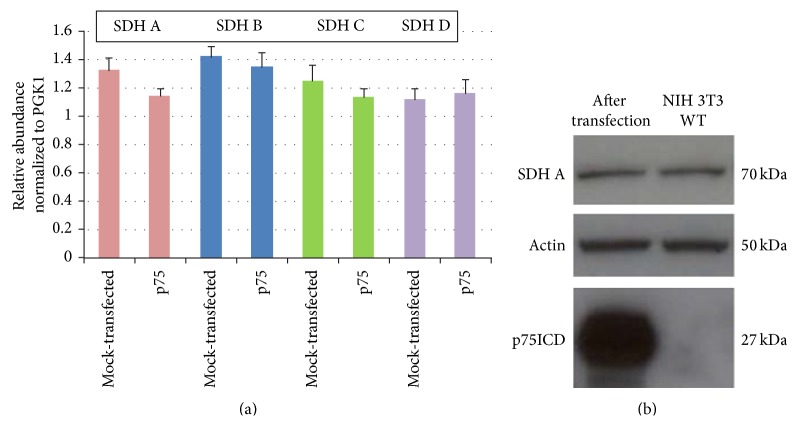
(a) qPCR for each of the subunits of Complex II (SDH A, B, C, and D) in p75ICD- or mock-transfected NIH 3T3 cells. Relative mRNA prevalence is shown from qPCR determination of SDH A, B, C, and D mRNAs from three independent experiments, each analyzing samples in triplicate. Error bars show SEM for the three experiments. (b) Western blot for SDH A in native (NIH 3T3 WT) and p75ICD-transfected NIH 3T3 cells confirms expression of p75ICD in the transfected cells.

We next asked whether induction of expression of p75ICD in NIH 3T3 cells alters expression of the four subunits of Complex II. All of the four subunits of this mitochondrial protein complex (i.e., SDH A, B, C, and D) are encoded by nuclear DNA.  Figure 2(a) shows the results of qPCR determinations of SDH A, B, C, and D mRNAs from three independent experiments each analyzing samples in triplicate. Induction of expression of p75ICD in NIH 3T3 cells did not result in a change in the level of mRNA for any of the four SDH subunits. We also determined by Western blotting the effect of induction of expression of p75ICD on the cellular concentration of SDH A protein. Induction of expression of p75ICD did not result in a change in SDH A protein (Figure 2(b)).

### 3.4. Effect of Induction of p75ICD or Its Fragments on Enzymatic Activity of Complex II

**Figure 3:**
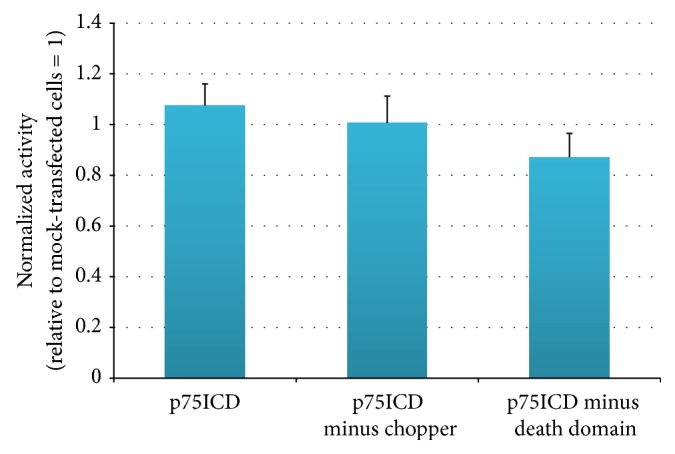
Complex II activity in NIH 3T3 cells transfected with expression constructs for p75ICD, p75ICD minus the chopper domain, or p75NTR death domain as described in [6] relative to activity in mock-transfected NIH 3T3 cells (set at 1.0). Complex II activity does not differ from that in mock-transfected cells for any of the p75ICD fragment transfectants (*p* > 0.05, Student's *t*-test). Error bars show SEM for three independent experiments, each with quadruplicate samples.

Finally, we examined the effects of induction of expression of p75ICD, p75ICD without the chopper domain, or the p75NTR death domain, respectively, on the cellular Complex II activity. Not surprisingly, there was no significant change in enzymatic activity of Complex II after induction of expression of p75ICD or its fragments by NIH 3T3 cells.  Figure 3 depicts the results of three independent experiments, each with samples studied in quadruplicate.

## 4. Discussion

Fenretinide is a retinoic acid analogue currently in clinical trials as a chemotherapeutic agent for neuroblastoma, a malignant tumor of childhood. It was originally developed as a potential differentiation-inducing agent for this tumor of the neural crest. However, its pharmacological activity is not dependent on retinoic acid receptor binding and it has been found to induce oxidative cell death via accumulation of mitochondrial reactive oxygen species [8]. We and others have demonstrated that fenretinide-induced mitochondrial oxidation and cell death are affected by 2-thenoyltrifluoroacetone, but not by rotenone or antimycin A, indicating that the mitochondrial reactive oxygen species are released at the level of Complex II [1, 8]. The generation of reactive oxygen species from Complex II requires the activity of 12-lipoxygenase and results in induction of GADD153, which, in turn, is required for oxidative induction of apoptosis [9, 10].

Our prior studies have also shown that expression of p75NTR enhances the oxidative and cytotoxic effects of fenretinide. p75NTR is a neurotrophin receptor signaling through which cleavage and nuclear trafficking of its intracellular domain are involved, thought to be a transcription factor [11]. The four subunits of mitochondrial Complex II are all encoded by nuclear DNA. We therefore hypothesized that enhancement by p75NTR of fenretinide-induced accumulation of mitochondrial reactive oxygen species from Complex II results from upregulation of the subunits of Complex II by p75ICD.

The present studies demonstrate that, while p75ICD does indeed traffic to the nucleus, neither p75ICD nor the fragments responsible for its activity induce enhanced mRNA or protein expression or enzymatic activity of Complex II in NIH 3T3 cells. Enhancement of the anticancer efficacy of fenretinide by p75NTR is therefore not the result of a direct effect of the transcription factor, p75ICD, on expression of the source of mitochondrial reactive oxygen species. Our previous studies have demonstrated that the effects of p75NTR on fenretinide-induced oxidation and cell death are dependent upon p38MAPK phosphorylation, JNK phosphorylation, caspase 3 activation, Akt cleavage, and decreased Akt phosphorylation [5]. In addition, treatment with fenretinide results in upregulation of p75NTR, MKK4, and MEKK1 and phosphorylation of MKK3/6 [5, 12]. Future studies aimed at identifying exploitable mechanisms through which efficacy of fenretinide against neuroblastoma and other cancers might best determine the relationship between these signaling effectors and mitochondrial accumulation of reactive oxygen species released at the level of Complex II.

## 5. Conclusion

Fenretinide is a chemotherapeutic agent in clinical trials for the treatment of neuroblastoma. The low bioavailability of this drug makes enhancement of bioavailability or efficacy imperative. Expression of p75NTR enhances fenretinide-induced oxidative cell death. But, despite the release from p75NTR of the transcription factor, p75ICD, this effect is not the result of enhanced expression of Complex II, an effector of the cellular activity of fenretinide.

## Figures and Tables

**Table 1:** Percent cell survival (MTS assay) of p75ICD- or mock-transfected NIH 3T3 cells treated with H_2_O_2_ and p75NTR- or mock-transfected SH-EP1 cells treated with fenretinide. In both cases, note the potentiation of the cytocidal effects of oxidative stress by induced p75NTR or p75ICD. Results represent mean ± SEM; *n* = 3 determinations at each concentration.

[H_2_O_2_]	0 *μ*M	200 *μ*M	600 *μ*M	1000 *μ*M
NIH 3T3 cells, p75ICD-transfected	100 ± 5	42 ± 1	21 ± 2	0 ± 0.2
NIH 3T3 cells, mock-transfected	100 ± 6	90 ± 5	85 ± 2	45 ± 5

[Fenretinide]	0 *μ*M	6 *μ*M	12 *μ*M	18 *μ*M

SH-EP1 cells, p75NTR-transfected	100 ± 1	88 ± 2	50 ± 1	38 ± 0.5
SH-EP1 cells, mock-transfected	100 ± 1	97 ± 1	94 ± 1	94 ± 0.8
